# Sodium Current Reduction Unmasks a Structure-Dependent Substrate for Arrhythmogenesis in the Normal Ventricles

**DOI:** 10.1371/journal.pone.0086947

**Published:** 2014-01-28

**Authors:** Patrick M. Boyle, Carolyn J. Park, Hermenegild J. Arevalo, Edward J. Vigmond, Natalia A. Trayanova

**Affiliations:** 1 Institute for Computational Medicine, Department of Biomedical Engineering, Johns Hopkins University, Baltimore, Maryland, United States of America; 2 Institut LIRYC, Université Bordeaux 1, Bordeaux, France; Brigham & Women’s Hospital - Harvard Medical School, United States of America

## Abstract

**Background:**

Organ-scale arrhythmogenic consequences of source-sink mismatch caused by impaired excitability remain unknown, hindering the understanding of pathophysiology in disease states like Brugada syndrome and ischemia.

**Objective:**

We sought to determine whether sodium current (I_Na_) reduction in the structurally normal heart unmasks a regionally heterogeneous substrate for the induction of sustained arrhythmia by premature ventricular contractions (PVCs).

**Methods:**

We conducted simulations in rabbit ventricular computer models with 930 unique combinations of PVC location (10 sites) and coupling interval (250–400 *ms*), I_Na_ reduction (30 or 40% of normal levels), and post-PVC sinus rhythm (arrested or persistent). Geometric characteristics and source-sink mismatch were quantitatively analyzed by calculating ventricular wall thickness and a newly formulated 3D safety factor (SF), respectively.

**Results:**

Reducing I_Na_ to 30% of its normal level created a substrate for sustained arrhythmia induction by establishing large regions of critical source-sink mismatch (SF<1) for ectopic wavefronts propagating from thin to thick tissue. In the same simulations but with 40% of normal I_Na_, PVCs did not induce reentry because the volume of tissue with SF<1 was >95% smaller. Likewise, when post-PVC sinus activations were persistent instead of arrested, no ectopic excitations initiated sustained reentry because sinus activation breakthroughs engulfed the excitable gap.

**Conclusion:**

Our new SF formulation can quantify ectopic wavefront propagation robustness in geometrically complex 3D tissue with impaired excitability. This novel methodology was applied to show that I_Na_ reduction precipitates source-sink mismatch, creating a potent substrate for sustained arrhythmia induction by PVCs originating near regions of ventricular wall expansion, such as the RV outflow tract.

## Introduction

The effects of macroscopic structure on impulse propagation in cardiac tissue are well understood, having been characterized in tissue wedges and geometrically simple computer models [1]–[4]. Wavefront propagation from thin to thick regions fails when source current emerging from the excited tissue is insufficient to elicit action potentials in non-excited downstream cells, which act as a current sink [5]. This source-sink mismatch at sites of structural expansion is exacerbated by conditions that impair tissue excitability, such as reduced depolarizing sodium current (I_Na_) density [2]. Establishing mechanistic understanding of how decreased excitability diminishes propagation robustness and leads to arrhythmogenic conduction block is of major significance because many conditions that cause sudden cardiac death are associated with decreased I_Na_, including ischemia [6] and Brugada syndrome (BrS) [7], [8]. Likewise, identifying links between source-sink mismatch and regions of wall thickness change inherent to heart structure may help explain why most BrS-related arrhythmias originate from ectopic excitations in the right ventricular outflow tract (RVOT) [9], [10], where the thinnest part of the RV connects to the thick left ventricular (LV) wall and the septum.

This study aims to test the hypothesis that global reduction of I_Na_ unmasks an arrhythmogenic substrate in the structurally normal ventricles by promoting source-sink mismatch in regions of thin-to-thick tissue expansion, such as the RVOT. To achieve our goal, we conduct simulations in an anatomically- and biophysically-detailed rabbit ventricular model with different degrees of I_Na_ reduction. We systematically characterize vulnerability to reentry induction by ectopic stimuli from several locations using a novel, quantitative evaluation of source-sink mismatch distribution in the whole heart.

## Materials and Methods

### Rabbit Ventricular Model

Reentry induction experiments were conducted in a detailed model of the rabbit ventricles incorporating realistic geometric structure and fiber orientations [11]. We chose this model because rabbit and human have comparable cardiac electrophysiological properties [12], but modeling smaller hearts is associated with a lower computational expense, which allowed us to perform an extensive number of simulations. Construction and validation of this simulation platform are described in our previous publications [13]–[15]. The resolution of the mesh described in those studies was increased twofold using tetrahedral subdivision to ensure numerical convergence (see Table S1 in File S1). Membrane kinetics in ventricular myocytes were represented by the rabbit-specific Mahajan-Shiferaw action potential (AP) model [16] and excitation propagation in tissue was governed by the monodomain formulation [17]. We conducted simulations under two electrophysiological conditions representing different degrees of global I_Na_ loss-of-function (30% or 40% of normal I_Na_). Other ionic current heterogeneities were deliberately omitted since our aim was to explore effects of global I_Na_ reduction in the absence of other potentially arrhythmogenic factors. Additional model parameters, including anisotropic tissue conductivities, are provided in Table S2 in File S1. All simulations were performed using the finite element method in the CARP software package [18].

### Simulation Protocol

For both electrophysiological conditions (30% and 40% of normal I_Na_), we pre-paced the ventricles with 10 sinus-like activations evoked by stimulating 53 LV and RV endocardial sites. We made this choice because closely coupled premature ventricular contractions (PVCs) are a well-known reentrant trigger, particularly in BrS patients [9]; sinus-like pre-pacing brought each ventricular model to a quasi-steady state and ensured a realistic repolarization sequence prior to each ectopic beat. Sinus stimulus locations and pacing times were chosen such that the resulting activation pattern in a simulation with normal I_Na_ (Fig. S1 in File S1) matched the excitation sequence for His stimulation, as we have described previously [19]. The basic cycle length between sinus beats was 400 *ms*, which is the baseline physiological heart rate for the rabbit ionic model [16]. Sinus stimulus duration and amplitude (10 *ms* at 60µ*A/cm^2^*) were adjusted to ensure capture at all 53 sites regardless of I_Na_ level.

We simulated PVCs by applying ectopic stimuli from 10 different endocardial locations. In keeping with our goal of examining source-sink mismatch in regions of wall expansion, most of these sites (7 of 10) were located near the insertion points of the RV into the LV, which is the most prominent example of disparity in tissue thickness in the structurally normal heart; sites E1–E4 were situated at points on the thin (RV) side of this structure, including the RVOT (E1), while E7–E9 were on the thick (LV) side. The remaining sites were located on the RV (E5–E6) and LV free walls. The coupling interval (CI) of each PVC was calculated with respect to the time from the start of the preceding sinus beat. All ectopic pacing stimuli lasted 10 *ms*. Stimulus strength was determined locally at each site by identifying the threshold to initiate AP propagation at that site in the simulation with 30% I_Na_ and CI = 300 *ms* then adding 20% to that value.

In simulations with 30% of normal I_Na_, we delivered ectopic stimuli from each of the 10 sites with CIs in the range 250–400 *ms* (5 *ms* intervals) and monitored for the establishment of sustained arrhythmia (reentry lasting ≥1000 *ms* after stimulus delivery). These experiments were conducted without post-PVC sinus stimuli (arrested sinus) to provide a long temporal window for analyzing source-sink mismatch. The same set of experiments was then repeated in simulations with 40% of normal I_Na_. Finally, a third set of simulations was performed with I_Na_ set to 30% and with continued sinus stimuli (basic cycle length of 400 *ms*) after each PVC (persistent sinus); the purpose of these experiments was to test whether such stimuli would initiate reentry or disrupt reentrant circuits observed in the initial (arrested sinus) protocol.

### Safety Factor

We developed a methodology to quantitatively evaluate the spatial distribution of possible source-sink mismatch in realistic 3D geometries during complex excitation sequences, such as ectopic beats. Our approach was based on a novel formulation for calculating cardiac safety factor (SF). SF was initially developed to quantify the robustness of AP propagation in 1D [20]; we recently introduced a new formulation suitable for calculating SF in higher dimensions [21]. The latter approach consisted of identifying the excitation interval (t_A_) during which upstream activity depolarized a particular downstream membrane patch; then, SF at that location was calculated as the ratio between the net charge delivered during this interval and the estimated threshold charge (Q_thr_) to elicit an AP by a stimulus of duration t_A_ in an isolated membrane patch in the same kinetic state.

This SF formulation, however, was insufficient for the present study for two reasons. First, the effect of CI was not accounted for in the calculation of Q_thr_. Second, the end of the t_A_ interval was defined as the instant when the total transmembrane current per unit area (I_m_) crossed below zero after an initial positive deflection; this condition (I_m_<0) does not always occur during sub-threshold excitation events in tissue with reduced I_Na_.

To address the first deficiency we added an explicit CI dependence to the Q_thr_ estimation using a two-step regression-based approach. The t_A_-dependent term Q_thr_(t_A_) in the original formulation was replaced by Q_thr_(t_A_,CI), defined as:
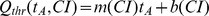
where m and b are CI-dependent slope and y-intercept functions, respectively. To derive these, we first used the process described in our earlier paper [21] to calculate linear-fit Q_thr_(t_A_) relationships for each CI in a wide time interval (200–800 *ms*, 5 *ms* increments; see Fig. S2A in File S1). Then, the resulting values were plotted versus CI and 6th-order least squares polynomial regression was used to obtain m(CI) and b(CI) (see Figs. S2B–C in File S1).

To address the original formulation’s failure to correctly identify t_A_ in some cases, we recast the entire SF calculation as a maximization problem over the interval between the local membrane depolarization (t_d_) and repolarization (t_r_) times:
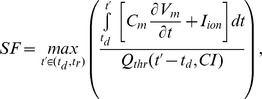
where t_d_ and t_r_ are the instants when the transmembrane voltage (V_m_) crossed above and below a threshold value (−85 *mV*), respectively, indicating transient perturbation from and return to resting V_m_; C_m_ is the specific membrane capacitance and I_ion_ is the ionic current per unit membrane area.

**Figure 1:**
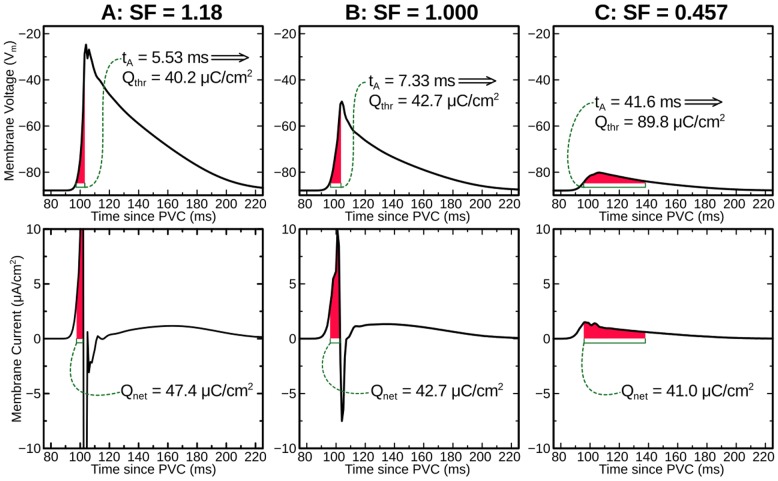
Graphical illustration of SF calculation from V_m_ and I_m_ traces in a model with 50% I_Na_ and arrested sinus rhythm. 3 cases are shown: just above threshold (A), at threshold (B), and well-below threshold (C). For a particular excitation interval t_A_, the charge necessary to initiate an AP is estimated based on behavior in uncoupled cells. Notably, for the example shown in (C), I_m_ never crosses below 0. SF: safety factor; V_m_: transmembrane voltage; I_m_: transmembrane current; I_Na_: sodium current; AP: action potential; Q_net_: charge available to depolarize the membrane (incoming minus outgoing).

With these improvements, our new SF formulation produced values that correctly indicated AP propagation robustness, even in cases where the I_m_<0 condition never occurred, as shown in Fig. 1. To facilitate reproducibility, pseudo-code for the above-described approach to SF calculation is provided in Appendix S1 in the File S1.

### Wall Thickness

Quantitative measurement of ventricular wall thickness (WT) allowed us to assess the effects of cardiac geometry on source-sink mismatch (as evaluated by the new SF formulation) and to identify structural characteristics common to locations from which ectopic stimuli were more likely to induce sustained reentry. We used a minimum distance algorithm to measure WT throughout the ventricles. First, surface points were manually categorized as epicardial, LV/RV endocardial, or LV/RV septal. Then, for each point in each set, the minimum distance to every other set was calculated; the smallest of these distances was selected as the local WT value. Using these values as a Dirichlet boundary condition, Laplace’s equation (i.e., 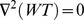) was solved on the ventricular domain to interpolate transmural WT values from epicardial and endocardial surface distributions, providing WT values throughout the ventricles.

## Results

**Figure 2:**
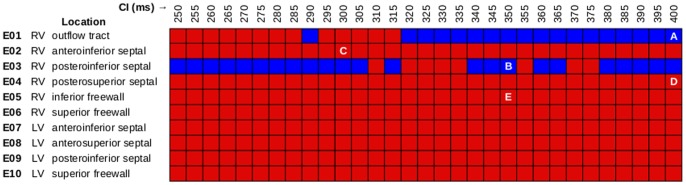
Vulnerability to induction of sustained arrhythmia by 310 ectopic stimuli from 10 different PVC sites (E1–E10) at 30% I_Na_ level and arrested sinus activation. See text for further detail. Specific simulations with activation patterns and/or V_m_ maps shown in Figs. 3, 5, or 6 are indicated by white text.

**Figure 3:**
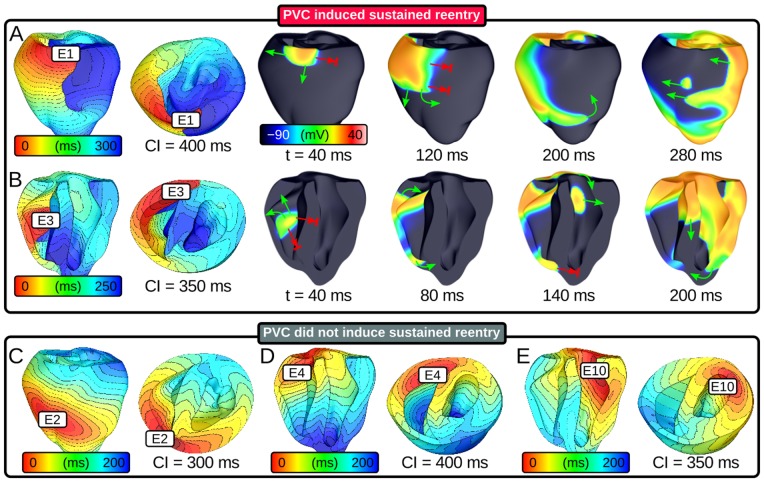
Response to ectopic stimulation in simulations with 30% I_Na_ and arrested sinus. (A–B) Activation sequences and V_m_ maps showing sustained arrhythmia induction by PVCs originating in two locations. Green arrows: successful wavefront propagation; red arrows: conduction block. (C–E) Activation sequences for three ectopic stimulation sites from which no episodes of sustained reentry were induced. Activation times measured relative to PVC delivery. Time scales vary from panel to panel but spacing between isochrones lines is always 10 *ms*. V_m_: membrane voltage; PVC: premature ventricular contraction.

**Figure 4:**
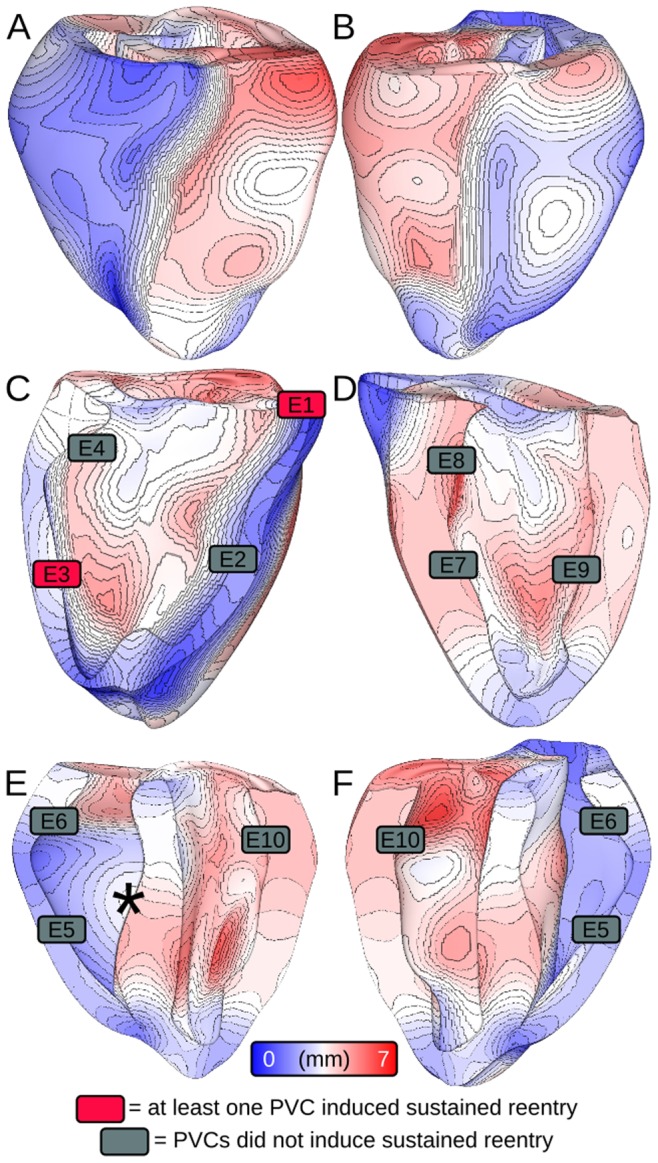
Maps of ventricular WT. (A–B) Anterior and posterior views. (C–D) Cutaway views of right and left sides of ventricular septum. (E–F) Cutaway views of posterior and anterior endocardium. All 10 endocardial PVC initiation locations are shown. Rectangle color indicates whether ectopic stimuli originating from that site induced sustained arrhythmia. Asterisk (in E) indicates a region of WT expansion in the RV free wall (see text for further detail). WT: wall thickness.

In accordance with the protocol described above, we ran 930 distinct simulations (10 sites ×31 CIs ×3 protocols) of closely coupled PVCs in the rabbit ventricular model and monitored for reentry induction. Outcomes for simulations with 30% of normal I_Na_ and arrested sinus rhythm are comprehensively presented in Fig. 2. Sustained arrhythmias were induced by ectopic stimuli originating from two of six RV sites and from zero of four LV sites. The regions from which PVCs initiated reentry were the RVOT (E1) and the RV posteroinferior septum (E3). The primary arrhythmogenesis mechanism in both cases was conduction block of wavefronts propagating from the thin RV into the thick LV and septum; activation sequences and spatiotemporal V_m_ maps for two reentry-inducing PVCs (Figs. 3A–B) illustrate this point. In the case of E1, there was a distinct line of transmural block along the RV-LV boundary extending from the base towards the apex (Fig. 3A and Movie S1). Conduction block leading to reentry induction also tended to occur very close to the site of ectopic activation and very soon after stimulus delivery, when the wavefront was still near the threshold for propagation failure; for the PVC originating from E3 (Fig. 3B and Movie S2), ectopic activity propagating into both the LV anterior septum and the more apical part of the RV free wall blocked almost immediately after the end of stimulation. This counter-intuitive conduction failure within the RV was due to a prominent bulge of the ventricular wall downstream from E3 in this part of the RV free wall (marked by an asterisk in Fig. 4E).

Ectopic activation sequences originating from three of the sites where PVCs never induced sustained reentry are shown in Figs. 3C–E. In all such cases, either the difference in thickness at the nearby wall expansion was insufficient to cause conduction failure or ectopic wavefronts were propagating robustly by the time they reached wall expansions. Thus, there were no instances of reentry induction, unlike in cases where PVCs occurred closer to regions with significant wall expansion (Figs. 3A–B). Notably, not all PVCs originating near the RV insertion into the LV initiated sustained reentry; wavefronts from E2 and E4 (Figs. 3C–D and Movies S3 and S4) always propagated into proximal LV and septal regions, in spite of considerably slowed conduction and even some instances of localized block.

To quantify our observation that sustained reentry-inducing PVCs involved conduction block in regions with steep ventricular wall expansion, we correlated ectopic stimulus locations with the corresponding WT values (Fig. 4). E1 was near a region of abrupt transition from thin RVOT (≈0.59 mm) to thick LV free wall (≈4.5 mm) and septum (≈5.8 mm); likewise, E3 was located near the posteroseptal RV insertion (≈1.8 mm), proximal to thick parts of the LV (≈4.2 mm) and septum (≈4.5 mm) as well as a bulge in the RV free wall (≈3.2 mm), which is marked by an asterisk in Fig. 4E. We also examined local WT values at RV sites from which PVCs never initiated sustained reentry. E4 was located in a relatively thick part of the RV (≈3.5 mm), which explained why ectopic wavefronts emerging from that site always activated the LV free wall and septum. The explanation for lack of reentry induction by PVCs from E2 was less straightforward – like E3, E2 was located in a thin part of the RV (≈1.5 mm), proximal to a thick part of the LV (≈4.6 mm) on the other side of the anterioseptal RV insertion; however, the septal region near E2 was much thinner (≈3.0 mm) than that near E3, which allowed ectopic activation from E2 to overcome slow conduction and activate the LV (see Fig. 3C).

**Figure 5:**
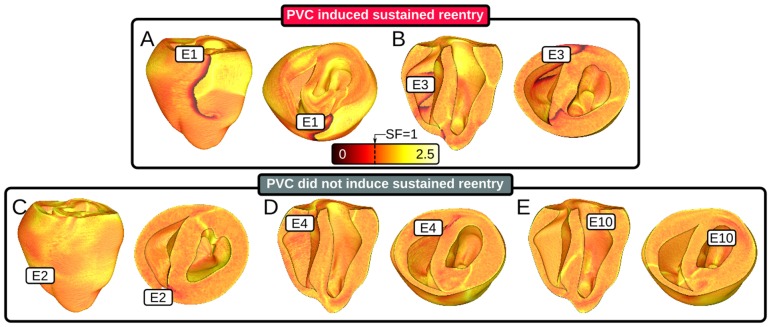
Maps of SF for propagation during the first excitation cycle following ectopic stimulation from several different sites in simulations with 30% of normal I_Na_. Thick lines of critical SF (black/red: SF<1) indicate locations of conduction block. (A–B) PVCs analyzed here induced sustained reentry (>1000 *ms*). Lines of critical SF were present along the septal insertion. (C–E) PVCs analyzed here did not induce reentry. SF: safety factor.

SF calculations showed quantitatively that source-sink mismatch markedly reduced the robustness of ectopic wavefront propagation from thin to thick ventricular wall regions (Fig. 5; panels A–E correspond to the same-lettered panels in Fig. 3). Locations of conduction block, seen as thick lines of SF <1, corresponded strikingly with closely-spaced iso-thickness lines in WT maps; for example, the SF calculation for E1 stimulation revealed conduction block along the RV-LV boundary, consistent with the activation sequence and V_m_ maps presented in Fig. 3A. Moreover, SF maps unmasked regions of less robust but non-failing propagation, such as the lower, thicker part of the RV free wall in the E1 case (SF≈1.1). In general, as the ectopic excitation front broadened and moved farther away from the site of PVC origination, propagation became more robust (SF≈1.5) due to decreased wavefront curvature. For sites from which ectopic stimuli never induced sustained arrhythmia (Fig. 5C–E), SF maps revealed incomplete lines of transmural conduction block. This confirmed that SF was still low for wavefronts propagating across wall expansions in these simulations, but not as low as in the cases of PVCs originating from the RVOT (E1) and posterior RV insertion (E3), where WT differences between the RV and both adjacent thicker walls were high (≥2.4 mm). As expected, SF of directly stimulated tissue was high (SF≈2.25).

**Figure 6:**
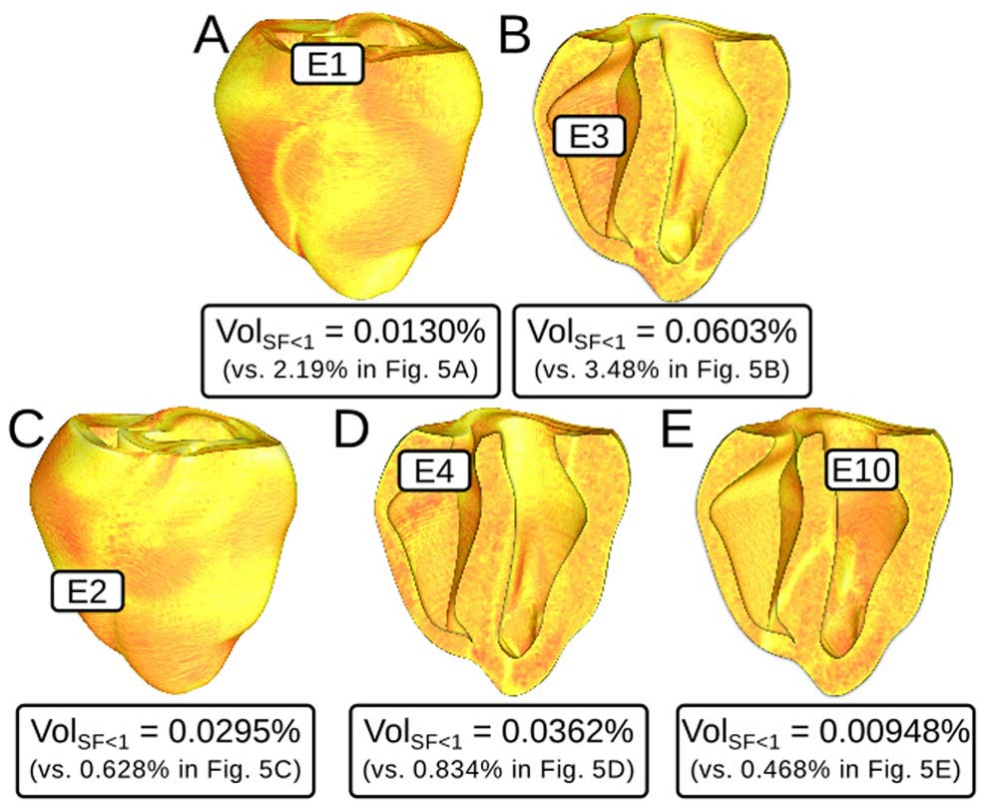
Maps of SF for propagation during the first excitation cycle following ectopic stimulation from several different sites in simulations with 40% of normal I_Na_ (same scale as Fig. 5); location and timing of all 5 PVCs is identical to Fig. 5. None of the PVCs analyzed here induced reentry. Labels show quantitative comparison to the corresponding SF maps in Fig. 5. Vol_SF<1_: percent volume of tissue in the ventricles with SF<1. Comparison of Vol_SF<1_ to the corresponding values in the simulations with 30% of normal I_Na_ shown in parentheses.

**Figure 7:**
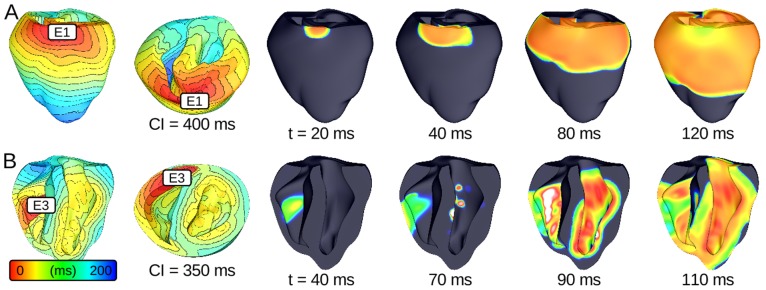
Activation sequences and V_m_ maps for PVCs that did not induce sustained arrhythmia. (A) Reentry was not induced due to higher I_Na_ (40% of normal); compare to Fig. 3A (same PVC in the simulation with 30% of normal I_Na_). (B) Persistent sinus activations prevented the induction of sustained arrhythmia; compare to Fig. 3B (same simulation with arrested sinus).

In simulations with 40% of normal I_Na_, there were no instances of reentry induction by PVC because source-sink mismatch was not as prominent. For the same PVCs analyzed in Fig. 5, the volume of tissue with SF<1 was at least 95% smaller, as shown in Fig. 6. For the case shown in Fig. 7A, the ectopic wavefront propagated from E1 into the LV and RV free walls and the septum; in contrast, for the same PVC under the 30% I_Na_ condition, weak conduction due to source-sink mismatch prevented excitation of the superior septum and LV free wall (see Fig. 3A). Movie S5 shows a side-by-side comparison of V_m_ sequences for these two simulations (Figs. 7A and 3A).

Similarly, in simulations with persistent sinus activation (and 30% of normal I_Na_), we observed no instances of sustained arrhythmia induction. This was because wavefronts from the first post-PVC sinus activation engulfed the excitable gap, eliminating conditions for reentry initiation. For the case shown in Fig. 7B, source-sink mismatch prevented LV activation by the ectopic stimulus, just as it did in the simulation of an identical PVC with arrested sinus (Fig. 3B); however, with persistent sinus, new wavefronts from sinus activation breakthrough sites (seen on the LV endocardium) prevented the establishment of reentry. Movie S6 shows a side-by-side comparison of V_m_ sequences for these two simulations (Figs. 7B and 3B).

## Discussion

The objective of this study was to determine whether I_Na_ loss-of-function directly increases the risk of sustained arrhythmia induction in structurally normal hearts by precipitating source-sink mismatch for wavefronts propagating from thin to thick ventricular wall regions. To achieve this goal, we simulated closely coupled PVCs in a biophysically detailed rabbit ventricular model. Our analysis was enabled by a newly developed approach for generating detailed 3D maps of SF, which allowed us to quantitatively assess source-sink mismatch throughout the ventricles. Key findings are as follows:

1. In the ventricular model with 30% of normal I_Na_ and arrested post-PVC sinus activations, ectopic stimulation induced sustained arrhythmia for a wide range of CIs when PVCs originated from sites where ectopic wavefronts propagated from thin to thick ventricular tissue, such as the RVOT;
2. Maps of SF, evaluated for the first time in the 3D ventricles, demonstrated that for reentry-inducing PVCs, critical source-sink mismatch (i.e., SF<1) occurred in areas of wavefront propagation from thin to thick ventricular wall causing conduction block, often near the ectopic stimulus site. Specifically, this behavior was observed for cases in which the LV-to-RV and septum-to-RV thickness differences were both ≥2.4 *mm*;
3. In the ventricular model with 40% of normal I_Na_, no PVCs induced reentry because >95% less tissue had SF<1 compared to 30% I_Na_ cases; and,
4. Persistent post-PVC sinus activations also reduced sustained arrhythmia inducibility because wavefronts from sinus sites engulfed the excitable gap, preventing reentry initiation.

These discoveries were made possible by the development of our novel methodology for calculating SF throughout the 3D ventricles, which allows the quantification of AP propagation robustness for ectopic wavefronts in tissue with impaired excitability and complex geometry. Our previous SF formulation suitably quantified the margin of safety for AP propagation in 2D tissue with homogeneous CI [21]; however, since ventricular activation sequences for PVCs differ from the sinus activation sequence, CI distribution is heterogeneous, necessitating the use of the new SF formulation developed here.

Our analysis of SF distribution in the ventricles and its correlation with ventricular wall thickness for nearly 1000 unique ectopic stimulation scenarios provides a detailed understanding of how reduced I_Na_ uncovers a substrate for reentry initiation due to inherently heterogeneous ventricular geometry in the absence of all other arrhythmogenic factors. In particular, we show mechanistically why PVCs originating near inherent thin-to-thick wall transitions in the structurally normal ventricles with impaired excitability are more likely to induce sustained arrhythmia compared to ectopic beats originating elsewhere. For these PVCs, I_Na_ reduction leads to a large region of critical source-sink mismatch (SF<1) along the boundary between the thin RV and the thick LV, which gives rise to conduction block. The fact that persistent post-PVC sinus activations preempted reentry initiation by consuming the excitable gap suggests that sustained arrhythmias could only be induced by this mechanism in the context of relatively slow heart rate or intermittent sinus rhythm (i.e., skipped beats). This is in agreement with clinical evidence, since BrS patients typically experience sustained arrhythmia and/or sudden cardiac death episodes during sleep, when the resting heart rate is lower [22], [23].

Previous studies used geometrically simplified models to examine source-sink mismatch in the context of reduced excitability, yielding the observation that decreased I_Na_ magnifies the electric load imposed upon the excitatory source (i.e., the wavefront) during impulse propagation from thin to thick tissue [2], [3]. The present study builds upon this classical work – in addition to exploring the dynamics of conduction failure in a geometrically detailed ventricular model, we also develop a new formulation for SF so that source-sink mismatch in the whole heart can be quantified. Since our SF formulation implicitly incorporates all underlying tissue properties, the same approach can be used in future studies to analyze how relevant electrophysiological heterogeneities affect impulse propagation and arrhythmia induction at the organ scale. For example, recent work has shown that impulse propagation in the RV is intrinsically more sensitive to I_Na_ availability compared to that in the LV [24], [25]; SF analysis in whole-heart models could be used to confirm our expectation that this electrophysiological heterogeneity exacerbates the arrhythmogenic effects of reduced excitability by further weakening ectopic sources originating in the RV. Additionally, several studies have reported shorter AP durations in the RV compared to the LV, leading to more homogeneous ventricular refractoriness between late-activating regions like the RVOT and the rest of the heart [26]–[28]. PVC timing did not play an obvious role in the arrhythmogenesis mechanism identified in this study, which suggests that effects due to refractoriness were minimal; nonetheless, it would be interesting to incorporate this type of LV/RV heterogeneity and apply the SF methodology developed here to achieve a more comprehensive understanding of reentry induction.

Insights revealed by this study regarding arrhythmogenic consequences of I_Na_ loss-of-function have important implications for understanding the pathophysiological basis of BrS. Due to the fact that the genetic basis of BrS is highly heterogeneous, many plausible theories have emerged to explain the underlying cause of disease-related arrhythmias [29]–[34]. However, since it seems unlikely that BrS symptoms can be solely attributed to any single factor, it has been suggested that individual mechanisms should be clarified and related to organ-level behaviors [35]. This paper leverages the flexibility of the computational platform and our new SF definition to present exactly that type of analysis – organ-scale arrhythmogenic effects of I_Na_ loss-of-function are examined in the absence of all other factors. The end result is a new explanation for the fact that ectopic beats arising in locations like the RVOT are much more likely to initiate reentry in BrS patients [9], [10].

In light of this finding, we speculate that a similar approach could be used to assess the organ-scale arrhythmogenic consequences of other electrophysiological abnormalities thought to be factors in BrS arrhythmogenesis. For instance, several studies have reported that I_Na_ in BrS has slower recovery from inactivation [36]–[39] and positively shifted steady-state activation [38], [40]. Moreover, BrS-associated mutations in other ion channels (decreased L-type calcium and increased transient outward potassium currents) were recently shown to mediate source-sink mismatch at tissue-scale heterogeneities (i.e., microscopic discontinuities between cell layers) [41]. Infiltration of the RV with non-excitable cells, such as fibroblasts [42] and/or adipocytes [43] has also been observed in BrS patients. Since all of the above factors could plausibly impair conduction safety and contribute to the inducibility of sustained arrhythmia by PVCs in BrS patients, investigation of their impact (individually or in concert) on organ-scale source-sink mismatch using the safety factor approach developed here would undoubtedly be a fruitful source of new understanding.

From a clinical standpoint, our findings suggest that cardiac imaging could be used to identify BrS patients with a high risk of experiencing arrhythmogenesis caused by source-sink mismatch in wall expansion regions. Between two individuals with the BrS genotype, we expect that the one with a particularly thin RVOT is more likely to experience a catastrophic episode of sustained arrhythmia initiated by this particular mechanism. Many studies have reported RV structural abnormalities in the hearts of BrS patients [42]–[45], but there has been no systematic analysis relating RV-to-LV thickness ratio to arrhythmogenesis. Some of the above-cited studies observed RV enlargement [42] and apparent thickening [43] but others reported subtle RVOT dilation [44], [45], which has been shown to coincide with regions of wall thinning [46]. This suggests that there must be a subset of BrS patients in whom the risk of arrhythmogenesis unmasked by source-sink mismatch, as identified in this paper, is even higher compared to other patients with BrS. Such mechanistic insights will help unravel the complex relations between BrS genotype and the disease manifestation.

## Supporting Information

File S1
**Supporting figures, tables and appendix. Figure S1:** Anterior (A) and left-lateral (B) views of activation sequence in response to sinus stimulation at t = 0 in the rabbit ventricular model (normal I_Na_); see Methods section for further details. Compare this excitation pattern to Fig. 3C in an earlier paper (reference [19]), which shows the sequence resulting from activation of the ventricles by a detailed model of the Purkinje system. I_Na_: cardiac sodium current. **Figure S2:** (A) Examples of Q_thr_(t_A_) functions derived by linear interpolation for different CI values showing differences in slope and y-intercept (r^2^>0.99 in all cases). (B & C) Slope and y-intercept values from panel A (gold squares), along with many others (red squares), are used to derive estimator functions for Q_thr_(t_A_,CI), as explained in Methods section. All values shown here were generated from single-cell simulations with 30% I_Na_. Sixth-order polynomial fits for these data sets (blue; r^2^>0.99) are given by the following two equations: 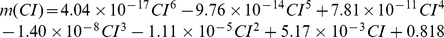 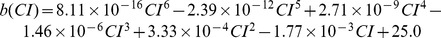 where Q_thr_ is the threshold charge to elicit an action potential in an uncoupled cell; t_A_ is the excitation interval (stimulus duration); and CI is coupling interval. **Table S1:** Convergence analysis conducted in a tissue wedge (5 cm×1 mm×1 mm) with the same electrophysiological properties as described in the Materials and Methods section. Simulations conducted with 30% of normal I_Na_. **Table S2:** Additional simulation details for rabbit ventricular models. **Appendix S1:** Pseudo-code for calculating safety factor according to the method described in this paper.(PDF)

Movie S1 **Anterior epicardial (left) and basal (right) views of sustained arrhythmia initiation by a PVC originating from site E1 in a simulation with 30% of normal I_Na_ and arrested sinus rhythm.** Maps show transmembrane voltage (V_m_) on the surface of the ventricles; same scale as Fig. 3A.
(MOV)

Movie S2 **Posterior endocardial cutaway (left) and basal cutaway (right) views of sustained arrhythmia initiation by a PVC originating from site E3 in a simulation with 30% of normal I_Na_ and arrested sinus rhythm.** Same scale as Movie S1.
(MOV)

Movie S3 **Anterior epicardial (left) and basal cutaway (right) views for a PVC originating from site E2 that did not induce sustained arrhythmia.** Same scale as Movie S1.
(MOV)

Movie S4 **Same views as Movie S2 for a PVC originating from site E4 that did not induce sustained arrhythmia.** Same scale as Movie S1.
(MOV)

Movie S5 **Side-by-side anterior epicardial view comparison of response to PVCs originating from E1 in simulations with 40% (left) or 30% (right) of normal I_Na_; right-hand panel is the same as left-hand panel from Movie S1.** Same scale as Movie S1.
(MOV)

Movie S6 **Side-by-side endocardial posterior cutaway view comparison of response to PVCs originating from E3 in simulations with persistent (left) or arrested (right) sinus activations; right-hand panel is the same as left-hand panel from Movie S2.** Same scale as Movie S1.
(MOV)
