# Supplementary material for: Sodium Current Reduction Unmasks a Structure-Dependent Substrate for Arrhythmogenesis in the Normal Ventricles
Source: PLoS One. 2014 Jan 28;9(1):e86947. doi: 10.1371/journal.pone.0086947 (PMC3904970; doi:10.1371/journal.pone.0086947)
Supplement: File S1 — Supporting figures, tables and appendix. Figure S1: Anterior (A) and left-lateral (B) views of activation sequence in response to sinus stimulation at t = 0 in the rabbit ventricular model (normal INa); see Methods section for further details. Compare this excitation pattern to Fig. 3C in an earlier paper (reference [19]), which shows the sequence resulting from activation of the ventricles by a detailed model of the Purkinje system. INa: cardiac sodium current. Figure S2: (A) Examples of Qthr(tA) functions derived by linear interpolation for different CI values showing differences in slope and y-intercept (r2>0.99 in all cases). (B & C) Slope and y-intercept values from panel A (gold squares), along with many others (red squares), are used to derive estimator functions for Qthr(tA,CI), as explained in Methods section. All values shown here were generated from single-cell simulations with 30% INa. Sixth-order polynomial fits for these data sets (blue; r2>0.99) are given by the following two equations: where Qthr is the threshold charge to elicit an action potential in an uncoupled cell; tA is the excitation interval (stimulus duration); and CI is coupling interval. Table S1: Convergence analysis conducted in a tissue wedge (5 cm×1 mm×1 mm) with the same electrophysiological properties as described in the Materials and Methods section. Simulations conducted with 30% of normal INa. Table S2: Additional simulation details for rabbit ventricular models. Appendix S1: Pseudo-code for calculating safety factor according to the method described in this paper. (PDF) [file pone.0086947.s001.pdf]

Sodium current reduction unmasks a structure-dependent substrate for arrhythmogenesis in the normal ventricles

Patrick M. Boyle[1†], Carolyn J. Park[1†], Hermenegild J. Arevalo[1], Edward J. Vigmond[2], Natalia A. Trayanova[1*]

1: Institute for Computational Medicine, Department of Biomedical Engineering, Johns Hopkins University, Baltimore, USA.
2: Institut LIRYC, Université Bordeaux 1, Bordeaux, France.

†: The first two authors contributed equally to this work.

*: Corresponding author: NA Trayanova, Phone: 410-516-4375, Fax: 410-516-5294, Email: ntrayanova@jhu.edu Mail: 3400 N Charles Street – 316 Hackerman Hall – Baltimore MD 21218 – USA

SUPPORTING FILE S1

Figure S1:

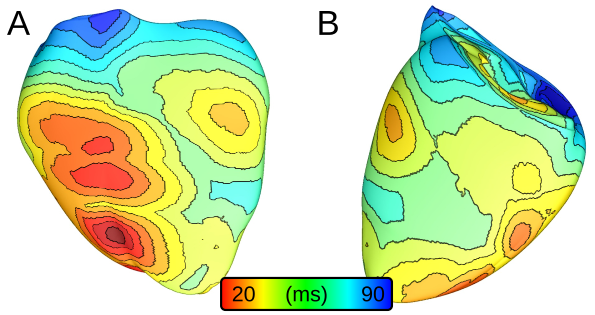

Figure S2:

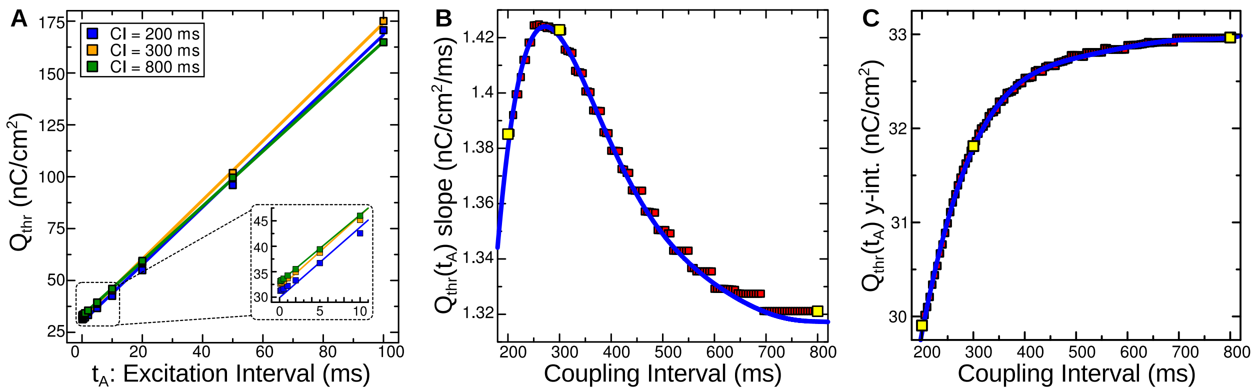

Table S1:

**Mesh Resolution (*μm*)**	**Conduction Velocity Along Wedge (*cm/s*)**	**Percent Difference (compared to highest-resolution model)**
500	19.24	42.5%
250	30.60	8.79%
125	33.09	1.37%
62.5	33.55	N/A

Table S2:

**Category**	**Parameter(s)**	**Value**
Geometry	# of degrees of freedom (nodes)	843,507
	# tetrahedral elements	4,744,512
	mean element edge length	261.76 *μm*
Monodomain conductivity (mS/mm)	longitudinal (σ_{mL})	0.1845
	transverse (σ_{mT})	0.02376
	normal (σ_{mN})	0.02376
Computation	# of Intel X5660 CPUs @ 2.80 *GHz*	12
	wall time to simulate 1 second	2*h*07*m*

Appendix:

```
################################################################
# Pseudocode for calculation of cardiac safety factor (SF)     #
# as described in Boyle, Park et al. (2013).                   #
#                                                              #
# This is an improved version of the formulation presented in  #
# Boyle & Vigmond (2010) Biophys J. Note that a correction to  #
# that paper was published in the December 2013 issue of the   #
# same journal (please see: http://goo.gl/DdHz0e).             #
#                                                              #
# The process outlined here is for a single node but can be    #
# extended to deal with arbitrary geometry, as in the paper.   #
################################################################

BEGIN CalcSF

    SET Vm   = read_file( Vm_file )           # transmembrane voltage data over time
    SET Iion = read_file( Iion_file )         # ionic current data over time

    SET dt = 0.1                              # global time step between data values
    SET last_act = ...                        # the previous activation time for this node

    # First, compute Im = Cm dVm/dt + Iion:
    SET Im = compute_Im( Vm, Iion, dt )
    # Note 1: current units: uA/cm^2, voltage units: mV
    # Note 2: reduces to a simple sum since Cm = 1 uF/cm^2
    # Note 3: use forward differencing to compute dVm/dt

    # Second, identify the first interval during which Vm is
    # above some empirically-defined threshold, e.g. -85 mV:
    SET Vm_thr =  -85
    SET i_depol = -1
    SET i_repol = -1
    FOREACH Vm_i in Vm,
        IF i_depol < 0 AND Vm_{i-1} < Vm_thr AND Vm_i >= Vm_thr,
                SET i_depol = i
        END IF
        IF i_repol < 0 AND Vm_{i-1} >= Vm_thr AND Vm_i < Vm_thr,
                SET i_repol = i
        END IF
    END FOREACH
    # Note 4:  To avoid spurious Vm crossings, it may be necessary to validate by ensuring
    #          that Vm stays above/below V_thr for some length of time (e.g., 1 ms)

    # Finally, calculate SF as a maximization over the identified trans-threshold interval:
    set SFmax = -1
    FOREACH i FROM i_depol+1 TO i_repol,
        SET Qnet = integrate( Im, i_depol, i, dt )
        # Note 5: a simple numerical integrator is OK (e.g., rectangle/trapezoid methods)

        SET Qthr = Qthr_tA_CI( dt * (i - i_depol), dt*i_depol - last_act )
        # Note 6: see text + Fig. S2 for detailed information on estimating Qthr(tA,CI)

        IF Qnet / Qthr > SFmax,
                set SFmax = Qnet / Qthr
        END IF
    END FOREACH

    # All done!
    print SFmax

END CalcSF
```
